# Circulating Neutrophil-to-Lymphocyte Ratio Predicts Stroke-Associated Infection and Poststroke Fatigue Affecting Long-Term Neurological Outcomes in Stroke Patients

**DOI:** 10.1155/mi/5202480

**Published:** 2025-04-22

**Authors:** Lei Zuo, Leiyu Geng, Yujia Cao, Xin-yu Zhou, Wu Di, Yun Liu, Zhe Zhong, Dandan Liu, Zhengsheng Zhang, Fuling Yan

**Affiliations:** ^1^Department of Neurology, Affiliated ZhongDa Hospital, Medical school of Southeast University, Nanjing, Jiangsu Province, China; ^2^Department of Neurology, The First Affiliated Hospital of Kangda College of Nanjing Medical University/The Affiliated Lianyungang Hospital of Xuzhou Medical University, Lianyungang, Jiangsu, China

**Keywords:** acute ischemic stroke, long-term outcome, neutrophil-to-lymphocyte ratio, poststroke fatigue, stroke-associated infection

## Abstract

**Background:** Since peripheral leukocytes may contribute to the pathophysiology of stroke, the aim of this study was to elucidate the relationship between leukocytes and stroke outcomes and identify which leukocyte subtypes most accurately predict functional outcomes and poststroke fatigue (PSF) in stroke patients.

**Methods:** A total of 788 ischemic stroke patients within 72 h of onset of disease were admitted in our study. Stroke-associated infection (SAI) and PSF were evaluated according to diagnosis standards by a special neurologist. Analyses were performed using SPSS 23.0 and GraphPad Prism 10.0.

**Results:** Neutrophil-to-lymphocyte ratio (NLR) has discriminative power in predicting stroke outcome, and the area under the curve (AUC) of NLR to distinguish stroke outcomes was 0.689 (95% confidence interval, 0.646–0.732). Positive correlation was found between NLR levels and NIHSS score on admission (*r* = 0.2786, *p* < 0.001). Risk model for predicting stroke outcome was constructed using age, NIHSS, previous stroke history, triglycerides, glucose and hemoglobin levels, thrombolysis treatment, and NLR, with an AUC of 0.865. Patients who developed SAI and PSF both had significantly higher NLR levels at admission than those patients not diagnosed with SAI and PSF (*p* < 0.0001). A risk model was constructed to predict PSF based on parameters including age, NIHSS score, lipoprotein(a) and NLR, and an AUC of 0.751.

**Conclusions:** Higher NLR levels in the acute phase of stroke might indicate a higher incidence of SAI and PSF. Therefore, higher NLR is associated with a poor stroke prognosis.

## 1. Introduction

Acute ischemic stroke (AIS) affects millions of people annually across the world and accounts for the main cause of death in China [1, 2]. Leakage of the blood–brain barrier after stroke can globally facilitate adhesion and migration of peripheral leukocytes to the brain parenchyma, which interact with glial cells, boost remote brain inflammation, and worsen stroke outcomes [3]. Therefore, peripheral circulating leukocytes in the acute phase after stroke might be imperative for neuroinflammation which is associated with stroke complications and stroke outcomes.

Previous studies have explored the relationship between circulating inflammation factors and stroke outcomes. Neutrophils contribute to stroke morbidity by clogging capillaries, even after successful thrombolysis and recanalization of the occluded artery [4]. Higher leukocyte count, neutrophil count, and neutrophil-to-lymphocyte ratio (NLR) after intravenous thrombolysis, as well as their dynamic changes, have been proposed independently associated with the increased risk of stroke outcomes [5]. Similarly, higher NLR predicted stroke-associated pneumonia (SAP) in patients with AIS, which is helpful in identifying high-risk patients in time and guiding preventive antibiotic therapy [6]. Moreover, NLR was an independent predictor of symptomatic hemorrhagic transformation after AIS and showed good discriminatory power [7]. It has been reported that pro-inflammatory state (the rise of NLR) contributes to cerebral volume loss in the contralateral hemisphere [8], which might affect long-term outcomes after stroke. Increased systemic inflammation, especially for NLR is linked to the severity of cerebral edema early after reperfusion therapy in stroke [9]. Accordingly, easily obtained inflammatory markers convey early warning alerts for patients at risk of severe neurological complications with impacts on physical outcomes.

Despite affecting stroke complications in the acute phase, inflammatory factors might predict neuropsychiatric complications after stroke which are harmful for long-term outcomes. NLR is a predictor of poststroke delirium, even independent of the development of infectious complications after stroke [10]. Also, it has revealed that white blood cells (WBCs) and neutrophil counts may be independent predictors of poststroke depressive symptomology and partial mediators of the relationship between stroke severity and poststroke depression, indicating that a significant role for neutrophil counts in the development of poststroke depression [11]. Similarly, the rise of plasma hs-CRP levels at admission was associated with poststroke fatigue (PSF) 6 months after stroke, indicating that these alterations might predict the development of PSF in stroke patients [12], which could affect the long-term outcome of stroke.

Since peripheral leukocytes may contribute to the pathophysiology of stroke and may have an impact on ensuing brain damage, demonstrating the prognostic role of leukocytes is crucial for the prevention of stroke complications and improving stroke outcomes either physically or mentally in AIS patients. Nevertheless, most previous researchers ignored to consider the contribution of leukocytes to clinical outcomes physically and mentally in one cohort. Thus, the aim of this study was to elucidate the relationship between leukocytes and stroke outcomes and identify which leukocyte subtypes were most precise in predicted functional outcomes and PSF in stroke patients.

## 2. Method

### 2.1. Study Population

The study cohort included 788 ischemic stroke patients who were admitted to neurology department of Zhongda Hospital within 72 h of onset of disease from September 2018 to August 2019. While only 714 of them finished modified Rankin scale (mRS) assessment and 524 of them finished the fatigue scale for motor and cognitive functions (FSMCs) score assessment 1 year after stroke onset. The detailed flowchart is provided in Supporting Information Figure S1. Among these, 109 patients received intravenous thrombolysis. Patients were included if they met both of the following criteria: (i) a new-onset ischemic stroke confirmed by cerebral computed tomography (CT) or magnetic resonance imaging in combination with stroke symptoms; (ii) peripheral blood sampling on admission before any treatment within 24 h of admission. Exclusion criteria were as follows: pre-existing hematologic, inflammatory, or autoimmune diseases; current cancer; infections preceding stroke; use of immunosuppressive drugs or use of antibiotics less than 24 h before admission; more than 72 h between time of stroke onset and admission; unclear time of onset; early death and/or initiation of palliative care (<48 h after admission); and recent surgery history within 3 months.

Upon 1 year after stroke onset, a mRS was used to determine the outcome: those with mRS scores between 0 and 2 indicate good prognosis, while those with mRS scores between 3 and 6 indicate poor prognosis.

Other methods were depicted in supplemental material.

## 3. Results

### 3.1. Characteristics of All Involved Participants

In this study, 714 patients diagnosed with AIS met the selection criteria, among which 32.91% (*n* = 235) developed poor outcomes 1 (mRS ≥ 3) year after stroke occurrence (Table 1). The median baseline age was 67 years (interquartile range [IQR] 57–76) for the entire cohort and 79 years (IQR 68–85) for patients with poor outcomes. In univariate analysis, age, gender, vascular risk factors (smoking history, atrial fibrillation, previous stroke history, coronary heart disease), baseline stroke severity (NIHSS), TOAST subtype, and treatment with intravenous thrombolysis were associated with stroke outcome. Patients who developed into poor outcomes also had significantly higher levels of glucose, lipoprotein (a), WBC, neutrophil count, NLR, creatinine, and D-dimer upon admission. Furtherly, the levels of triglycerides, lymphocyte counts, and hemoglobin on admission were significantly lower in patients with poor outcomes (Table 1).

### 3.2. The Relationship Between Inflammatory Indicators and Stroke Outcome

**Figure 1:**
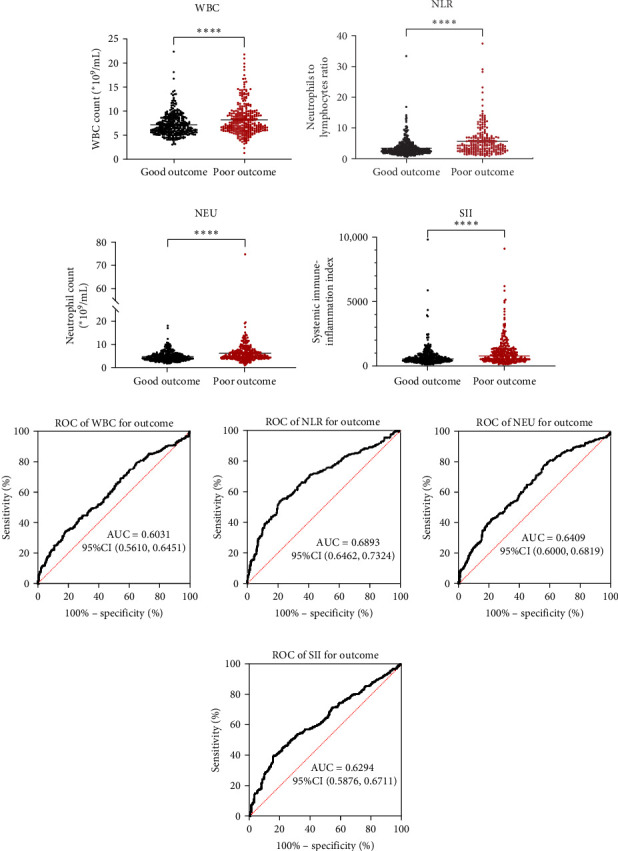
Relative levels of (A) WBC, (B) NLR, (C) neutrophil count, and (D) SIII in patients with good and poor outcomes. (E–H) indicates the Receiver operating characteristic (ROC) curves of them in predicting poor outcomes, respectively. NLR, neutrophil-to-lymphocyte ratio; NEU, neutrophil count; SIII, poststroke fatigue; WBC, white blood cells. *⁣*^*∗∗∗∗*^*p* < 0.0001, Mann–Whitney *U* test.

Taking those inflammatory indicators into consideration, the levels of WBC, neutrophil count, NLR and systemic immune-inflammation index (SII) all have predictive power for stroke outcome (Figure 1). As shown in Figure 1, the level of WBC, neutrophil count, NLR, and SII in poor outcome patients were significantly higher compared to those in good outcome patients (*p* < 0.0001 for all). Furthermore, the overall discriminative power of WBC, neutrophil count, NLR, and SII for outcomes was tested using receiver operating characteristic (ROC) curves, as shown in Figure 1. We found that the NLR's area under the curve (AUC) to distinguish between poor and good stroke outcomes was 0.689 (95%CI (confidence interval), 0.646–0.732), higher than the AUC of WBC (0.6031, 95%CI [0.5619, 0.6451]), neutrophil count (0.6409, 95%CI [0.6000, 0.6819]), and SII (0.6294, 95%CI [0.5876, 0.6711]). Based on the results above, NLR was hypothesized to independently predict outcomes. The optimal cutoff of NLR was defined as 4.155, with a sensitivity of 53.62% and a specificity of 78.85%.

### 3.3. Risk Model for Predicting Stroke Outcome

According to the possible prediction candidate factors found in univariate analysis, multiple logistic regression analysis was performed for predictors of stroke outcome, based on a stepwise MLRA (Backward Wald, Table 2). Due to missing values, the MLRA was performed on 644 of 714 patients. According to previous results in univariate analysis, the following variables were included: age, gender, smoking history, atrial fibrillation, previous stroke history, coronary heart disease, baseline NIHSS score, TOAST subtype, glucose level, triglycerides, lipoprotein (a), WBC, monocytes, neutrophils, lymphocytes, NLR, hemoglobin, creatinine, and D-dimer on admission and treatment with intravenous thrombolysis. Consequently, age, NIHSS, previous stroke history, triglycerides levels, glucose and hemoglobin levels, thrombolysis treatment, and NLR levels were independent predictors of poor outcomes after stroke (Table 2). Models 1 and 2 were based on independent predictors without or with NLR level. The AUC of the ROC curve Model 2 increased slightly to 0.865 compared to that of Model 1 (0.856) (Supporting Information Figure S2).

### 3.4. Peripheral NLR Correlates With Stroke Severity and Age

**Figure 2:**
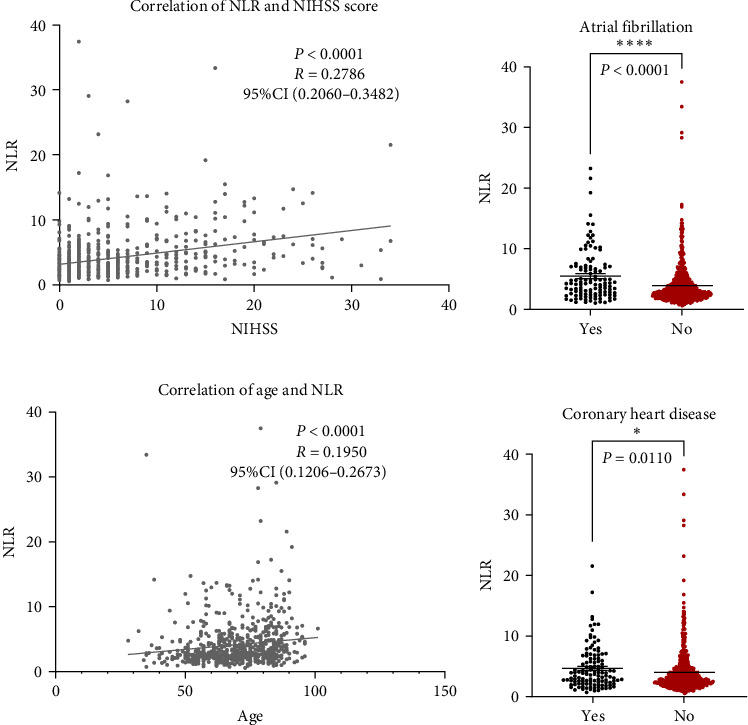
Relationship between neutrophil-to-lymphocyte (NLR) levels and stroke severity, age, and vascular risk factors. (A) Correlation between level of NLR and NIHSS score of stroke patients. *p* < 0.0001, Spearman analysis. (B) Correlation between NLR levels and stroke patient age. *p* < 0.0001, Spearman analysis. (C) Relative NLR levels in stroke patients with or without atrial fibrillation history, *⁣*^*∗∗∗∗*^*p* < 0.0001, Mann–Whitney test. (D) Relative NLR levels in stroke patients with or without coronary heart disease history, *⁣*^*∗*^*p* < 0.05, Mann–Whitney test.

NLR levels of the poor prognosis group were significantly higher than the good outcome group (*p* < 0.001, Table 1). Therefore, we further explored the relationship between peripheral NLR levels and stroke severity and age that has been reported to be associated with stroke outcomes. Positive correlation was found between NLR levels and NIHSS score on admission (*r* = 0.2786, *p* < 0.0001; Figure 2A) as well as patients' age (*r* = 0.1950, *p* < 0.0001; Figure 2B), indicating that higher NIHSS score and older age have corresponded to the higher NLR levels at admission. Moreover, statistical analysis revealed a significant influence of atrial fibrillation and coronary heart disease history on NLR levels (*p* < 0.0001 and *p*=0.0110 respectively, Figure 2C,D), while no influence of gender and vascular risk factors (hypertension, smoking history, diabetes mellitus, and previous stroke history) was found on NLR in stroke patients (*p* > 0.05, respectively, Supporting Information Figure S3).

### 3.5. The Relationship Between NLR and SAI

**Figure 3:**
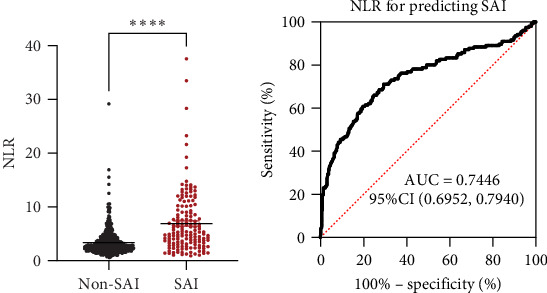
Relationship between neutrophil-to-lymphocyte (NLR) and stroke-associated infections (SAIs). (A) Relative NLR levels in stroke patients developed or not developed into SAI. *⁣*^*∗∗∗∗*^*p* < 0.0001, Mann–Whitney *U* test. (B) Receiver operating characteristic (ROC) of NLR in predicting SAI after stroke.

NLR has been verified to be a candidate biomarker for stroke-associated infection (SAI) which is a common complication after stroke and affects stroke outcome. Therefore, we tried to seek out whether NLR predicts SAI and further serves as a biomarker in our cohort. In order to further verify our hypothesis, we also found the level of NLR in SAI patients was significantly higher than that in non-SAI patients (Figure 3A, *p* < 0.0001). Further, ROC curves, which were depicted in Figure 3, were used to test the overall discriminative ability of NLR for SAI. We observed that the AUC of NLR to discriminate SAI was 0.7446 (95%CI, 0.6952–0.7440) (Figure 3B). The optimal cutoff of NLR was defined as 3.651, with a sensitivity of 71.15% and a specificity of 70.64%.

### 3.6. Prognostic Value of NLR in Thrombolysis and Nonthrombolysis Stroke Patients

While thrombolysis treatment might add confounding factors to the final outcome, we divided patients according to whether receive thrombolysis treatment or not. In this study, 109 patients received thrombolysis treatment, accounting for 15.27% of the present cohort. Regardless of whether thrombolysis treatment was received, the level of NLR has discriminative power in predicting stroke outcomes. The level of NLR is significantly higher in poor-outcome patients than in good-outcome patients (Supporting Information Figure S4A,C, *p* < 0.0001 for both). For patients who received rt-PA thrombolysis, the AUC of ROC cure was 0.6773 (95%CI, 0.5729–0.7817) (Supporting Information Figure S4B). The optimal cutoff of NLR was defined as 2.973, with a sensitivity of 73.91% and a specificity of 55.74%. While the prognostic power was not better than patients not receiving rt-PA thrombolysis. The AUC of ROC curve in patients not receiving rt-PA was 0.6969 (95%CI, 0.6496–0.7441) (Supporting Information Figure S4D). The optimal cutoff of NLR was defined as 4.155, with a sensitivity of 54.26% and a specificity of 80.05%.

### 3.7. The Relationship Between NLR Level on Admission and PSF

**Figure 4:**
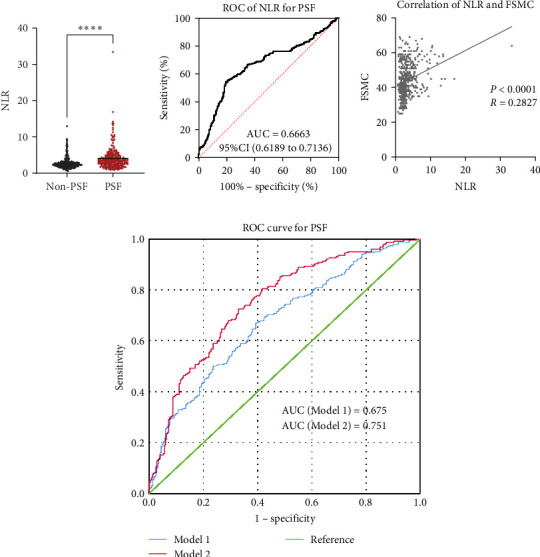
Relationship between neutrophil-to-lymphocyte (NLR) levels on admission and poststroke fatigue (PSF). (A) Relative NLR levels in stroke patients developed or not developed into PSF. *⁣*^*∗∗∗∗*^*p* < 0.0001, Mann–Whitney test. (B) Receiver operating characteristic (ROC) of NLR in predicting PSF. (C) Correlation between level of NLR and stroke patients' FSMC score. *p* < 0.0001, Spearman analysis. (D) ROC curve of logistic regression model for PSF. Model 1 is adjusted for age, NIHSS, and the level of Lpa and Model 2 was adjusted for NLR and parameters mentioned in Model 1. FSMC, fatigue scale for motor and cognitive function.

As for the long-term mental outcome, FSMC scores were measured 1 year after stroke onset. FSMC scores were measured by telephone or questionnaire evaluation, so those severe patients were unable to fulfil the questionnaire (mRS ≥ 4) and some patients were not willing to participate in FSMC follow-up. Therefore, only 524 patients were involved in the PSF evaluation. Among them, 258 stroke patients developed into PSF with FSMC score more than 43 and 266 stroke patients did not. The detailed information for stroke patients who developed into PSF or not develop into PSF has been listed in Table 3, and significant differences were found in age, NIHSS score, triglycerides, lipoprotein (a), WBC, neutrophil count, lymphocyte count, NLR, and D-dimer levels. Patients who developed PSF had significantly higher NLR levels at admission than those patients not diagnosed with PSF (Figure 4A, *p* < 0.0001). The AUC of the ROC curve (Figure 4B) based on NLR in predicting PSF was 0.6663 (95%CI, 0.6189–0.7136). Moreover, we further explored the relationship between peripheral NLR levels and FSMC score and found positive correlation between NLR levels and FSMC score (*r* = 0.2827, *p* < 0.0001; Figure 4C).

Based on candidate factors found in univariate analysis, multiple logistic regression analysis was performed to construct risk model of PSF (Backward Wald, Table 4). Consequently, age, NIHSS score, lipoprotein (a), and NLR have been validated to be independent risk factor of PSF. Models 1 and 2 have been constructed to predict the occurrence of PSF with and without depending on NLR, and the ROC of them have been shown in Figure 4D. The AUC of Model 2 (0.751) is significantly higher than that of Model 1 (0.675), further illustrating the significance of NLR in predicting PSF after stroke.

## 4. Discussion

In this study, we identified NLR as a valuable predictive marker for long-term stroke outcomes. Higher NLR levels might be associated with more severe symptoms, complicated by a higher incidence of SAI and more frequent occurrence of PSF. Therefore, higher NLR levels in the super-acute phase of stroke might reflect worse mental and physical recovery, indicating poor outcomes in long term.

It has been found that the changes in leukocytes occurring after stroke reflect the extent of brain injury as posited by the danger model of the immune response to specific and nonspecific brain damage (both ischemic and hemorrhagic stroke) [3]. Neutrophils, lymphocytes, and monocytes play complex interdependent roles that synergize to remove damaged brain tissue but also can cause subsequent injury to intact brain cells and generate maladaptive chronic inflammation [13]. Among peripheral leukocytes, neutrophils are imperative and have been shown to contribute to blood–brain barrier disruption in animal stroke models, thus exacerbating brain injury and leading to worse stroke outcomes [14]. On the contrary, different kinds of lymphocytes exert protection roles after stroke through their own pathways. T cells might exert hemostatic functions by their capacity to bind platelets through P-selectin and prevent hemorrhagic transformation after severe stroke [15]. B cells' neurotrophic potential permeated the poststroke brain and, in an antigen-independent way, produced early protection against ischemia injury [16]. Moreover, T regular cells actively suppressed autoimmune reactivity which served as one of the key mechanisms in preserving immune homeostasis and limiting inflammatory collateral damage [17]. Based on the mechanisms mentioned above, peripheral leukocytes have been verified to be associated with neuroinflammation and advocated as potential predictors of stroke outcomes in humans. Additionally, NLR has been verified to be more powerful in predicting stroke outcomes compared with the level of WBC, neutrophil count, and SII. Therefore, as a parameter resuming the information from both immune and adaptive immunity represents a reliable measure of the inflammatory burden [18], NLR has been used to evaluate the peripheral, possibly unspecific reaction to different types of brain damage.

As reperfusion therapy has been more and more widely used clinically, peripheral leukocytes might play more imperative roles in stroke patients receiving intravenous thrombolysis and mechanical thrombectomy. Therefore, neutrophil counts and NLR may serve as activity markers for parenchymal hemorrhage and 3-month poor prognosis in AIS patients who received intravenous thrombolysis [19]. Besides, another research based on stroke patients treated with mechanical thrombectomy found neutrophil and lymphocyte counts are dynamic parameters associated with hemorrhagic complications and long-term outcomes. The success of brain reperfusion and the extent of collateral circulation further influence the strength of these associations [20]. Therefore, NLR served as an independent predictor of 3-month mortality in AIS patients [21]. However, in this study, the significance of NLR in predicting stroke outcomes in patients receiving rt-PA thrombolysis is not better than that in patients not receiving rt-PA thrombolysis. Earlier blood collection time (within 4.5 h) and blood collection before the treatment of thrombolysis both might add influence of confounding factors. Thrombolysis treatment might have an impact on the peripheral inflammatory markers. Therefore, choosing the time for blood collection after initial treatment and limiting it as consistently as possible may be an important solution for further research.

Persistent fatigue is one of the most crippling symptoms of many neurological and mental disorders, including stroke. PSF is a common neuropsychological state which affects stroke outcome, too. Previous candidate biomarkers of PSF includes neuroimaging parameters [22, 23], stressful life events [24], inflammation factors [12], and emotional health. Inflammation indicators have been reported to be associated with psychological complications including PSF [12], poststroke depression [25], and poststroke delirium [10]. The reason why NLR might predict long-term PSF is complicated. The possible underlying mechanisms were listed as follows. The first hypothesis is related to mitochondrial dysfunction in brain caused by systemic inflammation in the acute phase, leading to energy deficiency sustained for long term [26]. Moreover, prolonged activation and chronic neuroinflammation of microglia caused by primary ischemic injury led to persistent neuronal dysfunction and impairments in neuronal homeostasis [27, 28]. Also, the elevation of NLR indicates the activation of proinflammatory cytokines, which exert negative effects on the production of neurotransmitters and induce fatigue through cytokine effects in brain areas involved in interception and homeostasis [29].

The main strengths of our study are not only evaluated the impact of NLR on stroke outcomes but also further examined how NLR predict long-term stroke outcomes. Poststroke complications and long-term mental illness have been reported to be associated with stroke outcomes, and thus we have evaluated the relation between NLR and SAI and PSF. Another strength is that through evaluation of the correlation between NLR and NIHSS score andFSMC score, we have investigated the role of NLR in predicting stroke severity and PSF by qualitative and quantitative analysis which makes the results more convincing.

Limitation includes the heterogeneity of involved stroke patients, leading to diversity treatment and comorbidities that might cause potential confounders. Patients who received thrombolysis were analyzed separately, making the prediction potential of NLR more reliable in this study. Moreover, patients involved in this study were within 72 h onset of stroke, while more closer restriction time might be better because the pathology and pathophysiology state changed a lot during this period. More time points should be considered in further research because dynamic changes of inflammatory indicators in the acute phase of stroke might be more valuable in predicting stroke outcomes [30]. Additionally, this study was only conducted in one clinic center, prospective validation in multicenter cohorts, and exploring dynamic changes in NLR during the acute and subacute phases of stroke were recommended for future research.

## 5. Conclusion

NLR has been verified to be a valuable predictive marker for long-term stroke outcomes. Higher NLR levels might be associated with more severe symptoms, complicated by a higher incidence of SAI and more frequent occurrence of PSF. Therefore, higher NLR levels in the super-acute phase of stroke might indicate the possibility of worse mental and physical recovery, suggesting poor outcomes in long term.

## Figures and Tables

**Table 1:** Baseline clinical characteristics in patients with different long-term outcomes.

	Favorable outcome	Unfavorable outcome	*p*
Demographic characteristics	—	—	—
Total, *n*	479	235	
Age, median (IQR), y	67 (57–76)	79 (68–85)	<0.001
Gender, female, *n* (%)	142 (29.65)	96 (40.85)	0.003
Vascular risk factors, *n* (%)	—	—	—
Hypertension	365 (76.20)	192 (81.70)	0.095
Smoking history	180 (37.58)	65 (27.66)	0.009
Atrial fibrillation	48 (10.02)	67 (28.51)	<0.001
Diabetes mellitus	175 (36.53)	90 (38.30)	0.647
Previous stroke	95 (19.83)	82 (34.89)	<0.001
Coronary heart disease	65 (13.57)	54 (22.98)	0.002
Toast subtype	—	—	<0.001
LAA	163 (34.03)	113 (48.09)	—
CE	27 (5.64)	48 (20.43)	—
SAO	243 (50.73)	45 (19.15)	—
ODC	46 (9.60)	29 (12.34)	—
Laboratory parameters, median (IQR)	—	—	—
Glucose (mmol/L)	5.72 (5.07–7.21)	6.48 (5.32–8.55)	<0.001
Hba1c	5.8 (5.3–6.9)	5.9 (5.5–7.0)	0.558
Triglycerides (mmol/L)	1.40 (1.01–2.13)	1.10 (0.81–1.56)	<0.001
Total cholesterol (mmol/L)	4.51 (3.79–5.19)	4.33 (3.60-5.09)	0.085
HDL (mmol/L)	1.15 (0.98–1.35)	1.15 (1.00–1.37)	0.537
LDL (mmol/L)	2.70 (2.12–3.25)	2.57 (1.90–3.28)	0.201
Lipoprotein(a) (mg/L)	141 (68–339)	196 (90–367)	0.043
WBC (10^9^/L)	6.73 (5.64–8.45)	7.49 (6.28–9.64)	<0.001
Monocytes	0.38 (0.29–0.48)	0.44 (0.32–0.56)	<0.001
Neutrophils (10^9^/L)	4.49 (3.58–5.73)	5.43 (4.19–7.61)	<0.001
Lymphocytes (10^9^/L)	1.62 (1.27–2.06)	1.25 (0.93–1.72)	<0.001
NLR	2.69 (2.02–3.97)	4.41 (2.67–6.88)	<0.001
Hemoglobin (g/L)	141 (130–153)	135 (120–146)	<0.001
Platelets (10^9^/L)	203 (166–240)	201 (162–238)	0.954
Creatinine (mmol/L)	72 (60–84)	76 (60–96)	0.007
D-dimer (ug/L)	122 (68–260)	327 (177–728)	<0.001
NIHSS, median (IQR)	2 (1–4)	8 (3–15)	<0.001
SAI, n (%)	67 (13.99)	90 (38.30)	<0.001
Thrombolysis, n (%)	63 (13.15)	46 (19.57)	0.025

Abbreviations: CE, cardioembolism; Cr, creatinine; HDL, high-density lipoprotein; IQR, interquartile range; LAA, large-artery atherosclerosis; LDL, low-density lipoprotein; NLR, neutrophil-to-lymphocyte ratio; ODC, stroke of other determined cause; SAI, stroke-associated infection; SAO, small-artery occlusion; WBC, white blood cell.

**Table 2:** Univariate logistic regression analysis for risk factors and risk model for stroke outcome.

	Model 1 without NLR		Model 2 with NLR	
	OR (95%CI)	*p*	OR (95%CI)	*p*
Age	1.052 (1.033–1.072)	<0.001	1.046 (1.026–1.067)	<0.001
NIHSS	1.266 (1.205–1.330)	<0.001	1.250 (1.189–1.315)	<0.001
Previous stroke	1.894 (1.208–2.970)	0.005	1.795 (1.136–2.838)	0.012
Triglycerides	0.746 (0.599–0.929)	0.009	0.768 (0.609–0.969)	0.026
Glucose	1.101 (1.018–1.191)	0.017	1.089 (1.003–1.183)	0.043
Hemoglobin	0.996 (.988–1.003)	0.245	0.984 (0.973–0.995)	0.006
Thrombolysis	0.434 (0.237–0.796)	0.007	0.437 (0.235–0.813)	0.009
NLR	—	—	1.108 (1.038–1.182)	0.002

**Table 3:** Baseline clinical characteristics in patients diagnosed or not diagnosed with PSF 1 year after stroke.

	Non-PSF	PSF	*p*
Demographic characteristics	—	—	—
Total, *n*	303	234	
Age, median (IQR), y	66 (57–73)	70 (59–79)	0.002
Female, *n* (%)	91 (30.03)	67 (28.63)	0.724
Vascular risk factors, *n* (%)	—	—	—
Hypertension	227 (74.92)	188 (80.34)	0.137
Smoking history	123 (40.59)	86 (36.75)	0.365
Atrial fibrillation	25 (8.25)	31 (13.25)	0.060
Diabetes mellitus	114 (37.62)	88 (37.61)	0.997
Previous stroke	62 (20.46)	51 (21.79)	0.707
CHD	37 (12.21)	36 (15.38)	0.287
Toast subtype	—	—	0.103
LAA	98 (32.34)	90 (38.46)	—
CE	14 (4.62)	19 (8.12)	—
SAO	158 (52.15)	102 (43.59)	—
ODC	33 (10.89)	23 (9.83)	—
Laboratory parameters, median (IQR)	—	—	—
Glucose (mmol/L)	5.65 (5.00–7.34)	5.76 (5.16–7.13)	0.483
Hba1c	5.8 (5.25–7.05)	5.85 (5.33–6.90)	0.518
Triglycerides (mmol/L)	1.47 (1.06–2.28)	1.30 (0.88–1.92)	0.012
Total cholesterol (mmol/L)	4.55 (3.77–5.19)	4.53 (3.74–5.25)	0.838
HDL (mmol/L)	1.15 (0.98–1.34)	1.16 (0.98–1.35)	0.911
LDL (mmol/L)	2.74 (2.14–3.31)	2.69 (2.07–3.28)	0.517
Lpa (g/L)	123 (59–307)	174 (79–412)	0.004
WBC (10^9^/L)	6.56 (5.56–8.00)	7.00 (5.87–8.87)	<0.001
Monocytes	0.39 (0.30–0.48)	0.39 (0.30–0.52)	0.056
Neutrophils (10^9^/L)	4.17 (3.40–5.24)	4.82 (3.89–6.39)	<0.001
Lymphocytes (10^9^/L)	1.74 (1.37–2.22)	1.44 (1.13–1.92)	<0.001
NLR	2.35 (1.85–3.00)	3.52 (2.49-–4.91)	<0.001
Hemoglobin (g/L)	143 (133–154)	141 (130–153)	0.284
Platelets (10^9^/L)	210 (177–248)	201 (164–236)	0.216
Cr (mmol/L)	72 (58–84)	71 (61–85)	0.690
D-dimer	112 (66–215)	153 (81–429)	<0.001
NIHSS, median (IQR)	2 (1–4)	3 (2–6)	<0.001
SAI, *n* (%)	36 (13.99)	44 (38.30)	0.025
Thrombolysis, *n* (%)	36 (13.15)	37 (19.57)	0.188

Abbreviations: CE, cardioembolism; Cr, creatinine; HDL, high-density lipoprotein; IQR, interquartile range; LAA, large-artery atherosclerosis; LDL, low-density lipoprotein; NLR, neutrophil-to-lymphocyte ratio; ODC, stroke of other determined cause; SAI, stroke-associated infection; SAO, Small-artery occlusion; WBC, white blood cell.

**Table 4:** Univariate Logistic regression analysis for risk factors and risk model for PSF

	Model 1 without NLR		Model 2 with NLR	
	OR (95%CI)	*p*	OR (95%CI)	*p*
Age	1.025 (1.009–1.040)	0.002	1.024 (1.008–1.041)	0.004
NIHSS	1.155 (1.089–1.225)	<0.001	1.131 (1.062–1.205)	<0.001
Lpa (g/L)	1.001 (1.000–1.002)	0.021	1.001 (1.000–1.001)	0.051
NLR	—	—	1.367 (1.216–1.537)	<0.001

## Data Availability

The data that support the findings of this study are available from the corresponding author upon reasonable request.

## Supporting Information

Supporting Information Supporting Information: Circulating neutrophil to lymphocyte ratio predict stroke associated infection and poststroke fatigue affecting long-term neurological outcomes in stroke patients. Figure S1: Flowchart showing total involved participants in present analysis. Figure S2: ROC curve of logistic regression model for stroke outcome. Model 1 is adjusted for age, NIHSS, previous stroke history, thrombolysis treatment and the level of triglycerides, glucose, hemoglobin, and Model 2 was adjusted for NLR and parameters mentioned in Model 1. Figure S3: Relationship between NLR levels and gender as well as vascular risk factors. (A) No significant difference of NLR level was found in stroke patients' gender. *p*=0.7887, Mann–Whitney test. (B) No significant difference of NLR level was found between stroke patients with or without smoking history. *p*=0.0948, Mann–Whitney test. (C) No significant difference of NLR level was found between stroke patients with or without previous stroke history. *p*=0.1746, Mann–Whitney test. (D) No significant difference of NLR level was found between stroke patients with or without hypertension history. *p*=0.4290, Mann–Whitney test. (E) No significant difference of NLR level was found between stroke patients with or without diabetes history. *p*=0.5876, Mann–Whitney test. Figure S4: Relative level of NLR in thrombolysis and non-thrombolysis stroke patients. (A) Relative level of NLR in good-outcome group and poor-outcome group for stroke patients received thrombolysis treatment. *⁣*^*∗∗*^*p* < 0.01, Mann–Whitney *U* test. (B) ROC of NLR in predicting outcome for stroke patients received thrombolysis treatment. (C) Relative level of NLR in good-outcome group compared to poor-outcome group for stroke patients not received thrombolysis treatment. *⁣*^*∗∗∗∗*^*p* < 0.0001, Mann–Whitney *U* test. (D) ROC of NLR in predicting outcome for stroke patients not received thrombolysis treatment.
