# Supplementary material for: Circulating Neutrophil-to-Lymphocyte Ratio Predicts Stroke-Associated Infection and Poststroke Fatigue Affecting Long-Term Neurological Outcomes in Stroke Patients
Source: Mediators Inflamm. 2025 Apr 22;2025:5202480. doi: 10.1155/mi/5202480 (PMC12041617; doi:10.1155/mi/5202480)
Supplement: Supporting Information — Circulating neutrophil to lymphocyte ratio predict stroke associated infection and poststroke fatigue affecting long-term neurological outcomes in stroke patients. Figure S1: Flowchart showing total involved participants in present analysis. Figure S2: ROC curve of logistic regression model for stroke outcome. Model 1 is adjusted for age, NIHSS, previous stroke history, thrombolysis treatment and the level of triglycerides, glucose, hemoglobin, and Model 2 was adjusted for NLR and parameters mentioned in Model 1. Figure S3: Relationship between NLR levels and gender as well as vascular risk factors. (A) No significant difference of NLR level was found in stroke patients' gender. p=0.7887, Mann–Whitney test. (B) No significant difference of NLR level was found between stroke patients with or without smoking history. p=0.0948, Mann–Whitney test. (C) No significant difference of NLR level was found between stroke patients with or without previous stroke history. p=0.1746, Mann–Whitney test. (D) No significant difference of NLR level was found between stroke patients with or without hypertension history. p=0.4290, Mann–Whitney test. (E) No significant difference of NLR level was found between stroke patients with or without diabetes history. p=0.5876, Mann–Whitney test. Figure S4: Relative level of NLR in thrombolysis and non-thrombolysis stroke patients. (A) Relative level of NLR in good-outcome group and poor-outcome group for stroke patients received thrombolysis treatment. ⁣∗∗p < 0.01, Mann–Whitney U test. (B) ROC of NLR in predicting outcome for stroke patients received thrombolysis treatment. (C) Relative level of NLR in good-outcome group compared to poor-outcome group for stroke patients not received thrombolysis treatment. ⁣∗∗∗∗p < 0.0001, Mann–Whitney U test. (D) ROC of NLR in predicting outcome for stroke patients not received thrombolysis treatment. [file 5202480.f1.docx]

**Supplementary Material**

**Circulating neutrophil to lymphocyte ratio predict stroke associated infection and post-stroke fatigue affecting long-term neurological outcomes in stroke patients**

**Methods**

**1. Data Collection**

Demographic data, medical history, baseline stroke severity (National Institutes of Health Stroke Scale, NIHSS) were collected on admission. Also, laboratory parameters were collected and recorded in detail including glucose level, HbA1c, Triglycerides, total cholesterol, high density lipoprotein(HDL) , low density lipoprotein(LDL), lipoprotein(a), white blood cells(WBC), neutrophil count, lymphocyte count, monocyte count, platelet count, hemoglobin, serum creatinine level(Cr), d-dimer level. NLR was defined as neutrophil count / lymphocyte count. Systemic immune-inflammation index(SIII) was defined as platelet count × neutrophil count/lymphocyte count. Classification of ischemic stroke subtypes was based on the Trial of Org 10172 in Acute Stroke Treatment criteria. Whether receive thrombolysis treatment or not was recorded too.

**2. Clinical assessment**

Before treatment, the patients’ NIHSS (National Institute of Health Stroke Scale) were assessed by certified stroke physicians. All the clinical characteristics were recorded and assessed upon admission, too.

Stroke associated infection (SAI)

SAI included two main complications after stroke-urinary tract infection (UTI) and SAP and were diagnosed within 7 days after stroke onset according to the modified criteria of the US Disease Control and Prevention Centers. The diagnosis of SAP was made when at least one of the former and one of the latter criteria were fulfilled: (1) pulmonary infiltrates on chest x-rays, abnormal respiratory examination; (2) productive cough with purulent sputum, microbiological cultures from lower respiratory tract or blood cultures, leukocytosis, elevation of C-reactive protein (CRP). UTI was diagnosed when at least 2 of the following criteria were fulfilled: fever (>38°C), urine sample positive for nitrite, leukocyturia, and significant bacteriuria.

Post-stroke fatigue evaluation

Fatigue severity was measured with the fatigue scale for motor and cognitive functions (FSMC) by standard telephone interview or reexamination at 1 year after stroke onset. Only those stroke patients with mRS<4 were involved in the PSF evaluation. The FSMC consists of 20 questions with response options rated on a five-point scale from “never” (1) to “always”(5), resulting in a total score from 20 (minimum score) and 100 (maximum score). Using FSMC score≥43 as definition of PSF. The higher the score, the greater the impact of fatigue on the patient’s daily life.

**3.Statistical Analysis**

Analyses were performed using SPSS 23.0 and GraphPad Prism 10.0 (USA). Differences between pairs were tested using the Mann–Whitney test. Statistical analysis for group comparisons was performed using the Kruskal–Wallis test, followed by a post hoc Dunn’s comparison test. Statistical analysis for qualitative variables was performed using Fisher’s exact test. Correlation analysis was performed using the Spearman test. The influence of NLR on stroke outcome and PSF were assessed using univariate and multivariate binary logistic regression analysis with significant confounding factors tested in the univariate analysis adjusted. Results were expressed as adjusted odds ratios (OR) with the corresponding 95% confidence intervals (CI). Receiver operating characteristic (ROC) curve analysis was utilized to evaluate the cut-off point on the NLR levels on admission with the greatest sensitivity and specificity to predict stroke outcome and post-stroke fatigue. Differences were considered statistically significant when P-value < 0.05.


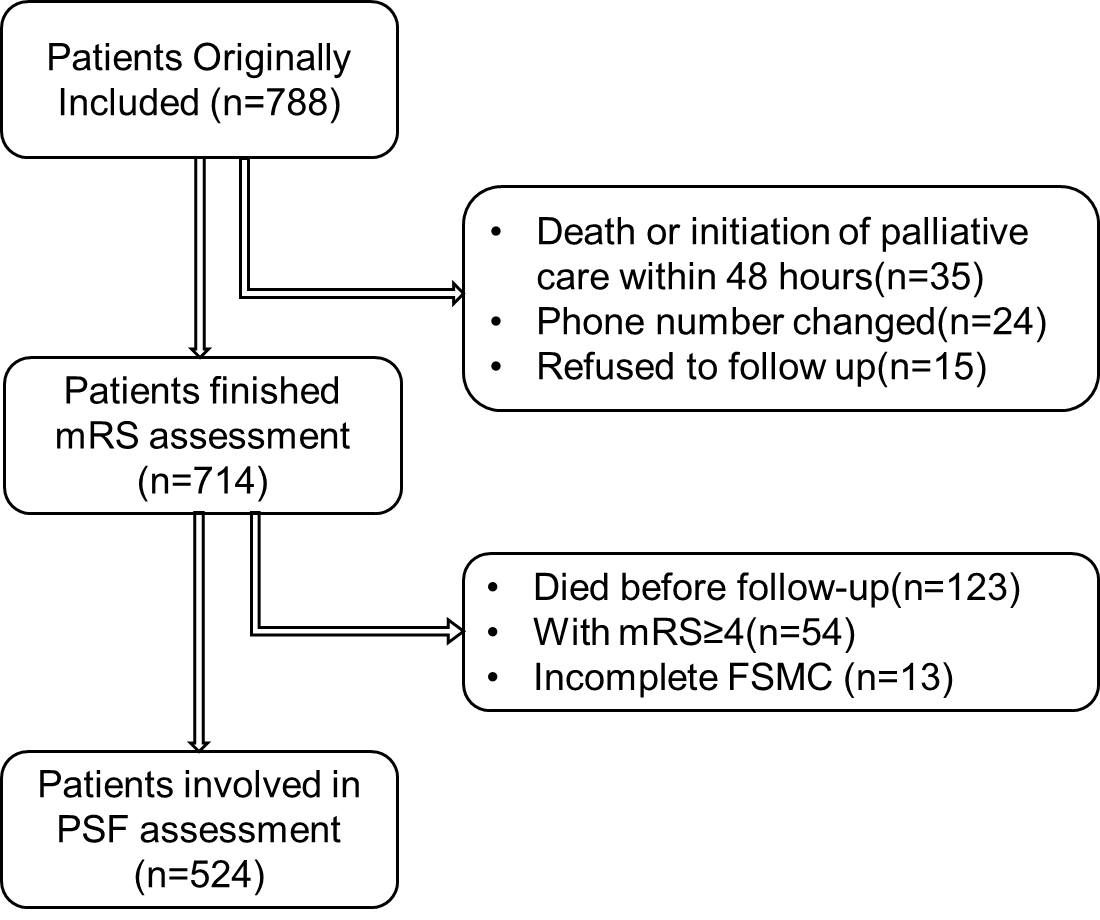


**Supplemental Figure 1.** Flowchart showing total involved participants in present analysis


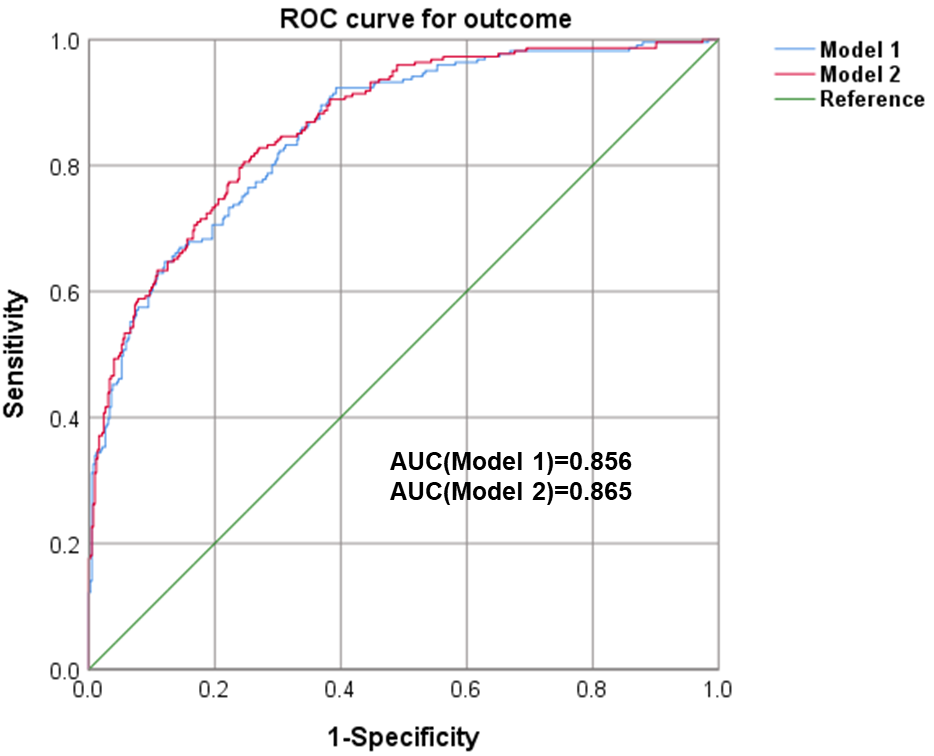


**Supplemental Figure 2**. ROC curve of logistic regression model for stroke outcome. Model 1 is adjusted for age, NIHSS, previous stroke history, thrombolysis treatment and the level of triglycerides, glucose, hemoglobin and Model 2 was adjusted for NLR and parameters mentioned in Model 1.


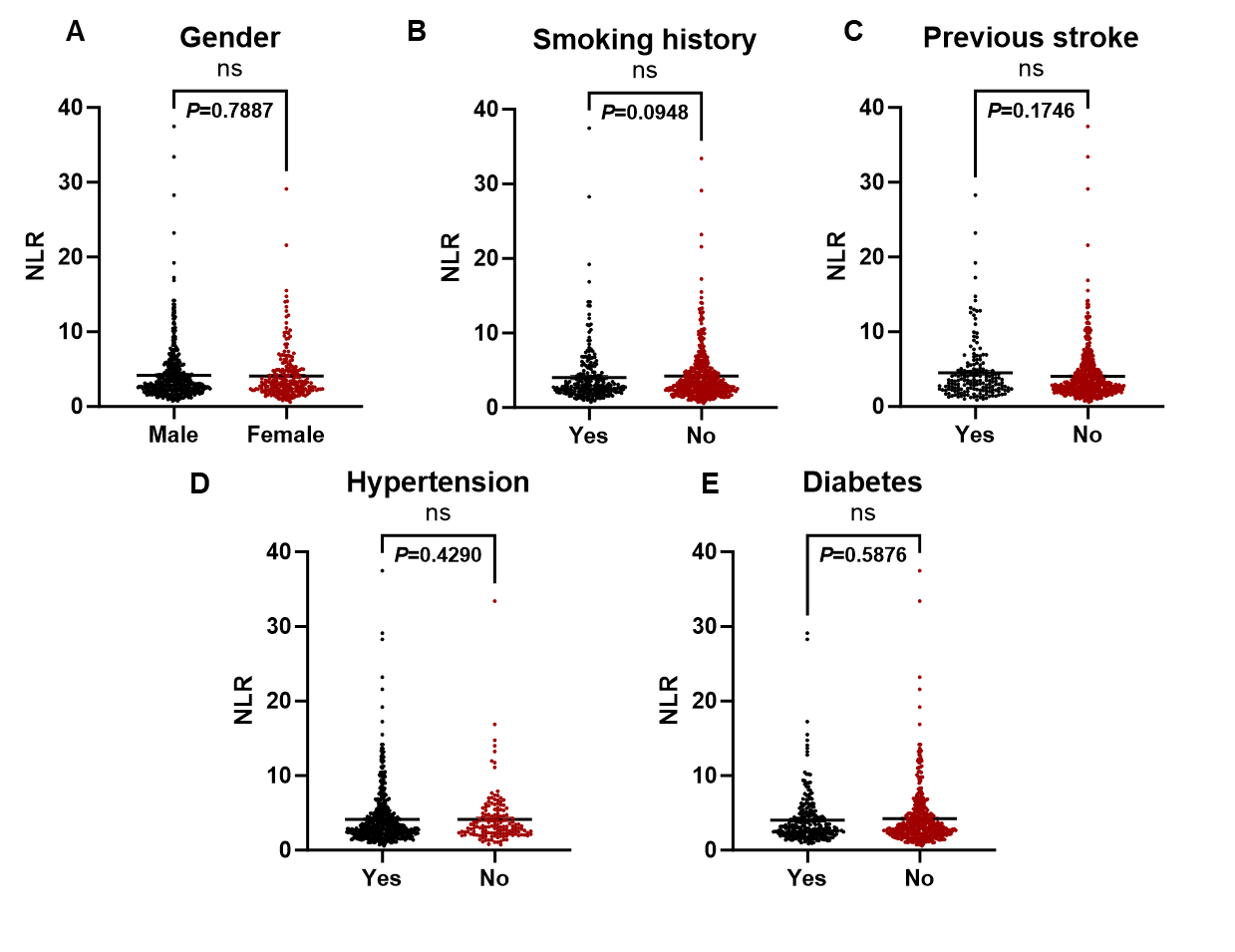


**Supplemental Figure 3**. Relationship between NLR levels and gender as well as vascular risk factors. (A) No significant difference of NLR level was found in stroke patients’ gender. P=0.7887, Mann–Whitney test. (B) No significant difference of NLR level was found between stroke patients with or without smoking history. P=0.0948, Mann–Whitney test. (C) No significant difference of NLR level was found between stroke patients with or without previous stroke history. P=0.1746, Mann–Whitney test. (D) No significant difference of NLR level was found between stroke patients with or without hypertension history. P=0.4290, Mann–Whitney test. (E) No significant difference of NLR level was found between stroke patients with or without diabetes history. P=0.5876, Mann–Whitney test.


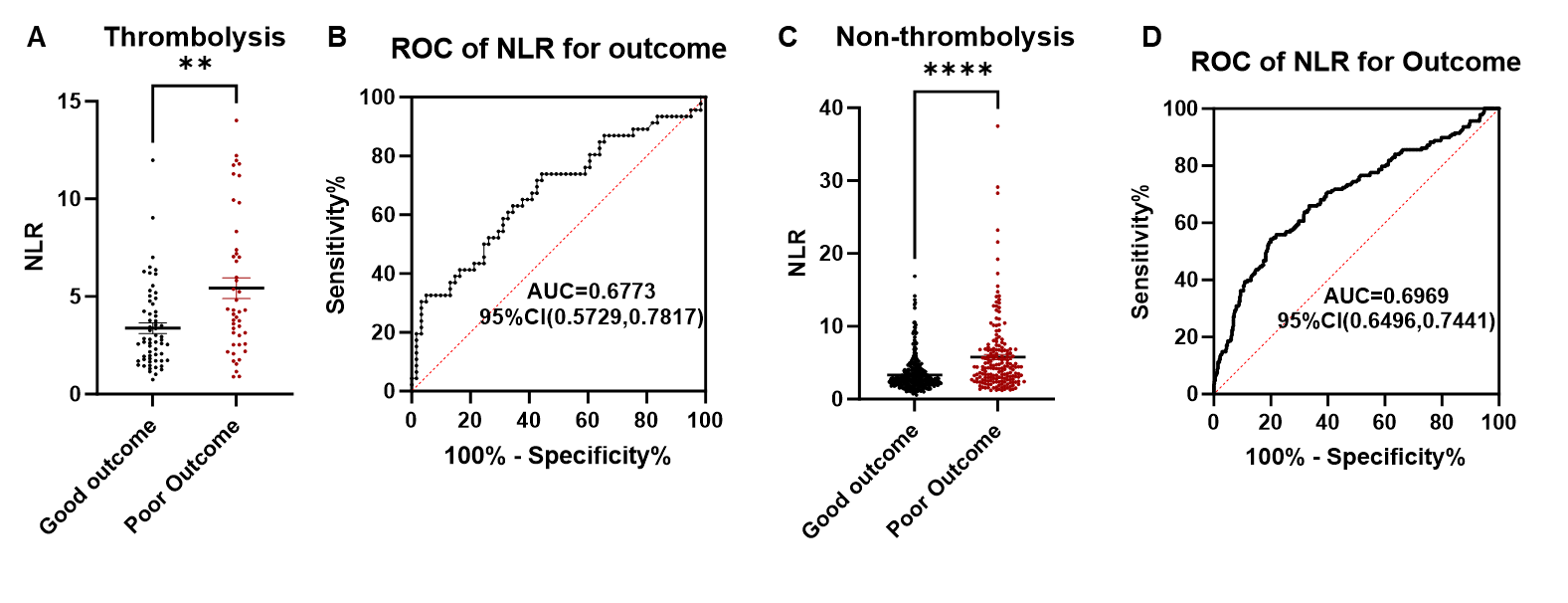


**Supplemental Figure 4.** Relative level of NLR in thrombolysis and non-thrombolysis stroke patients **(A)** Relative level of NLR in good outcome group and poor outcome group for stroke patients received thrombolysis treatment. **P<0.01, Mann-Whitney U test. **(B)** ROC of NLR in predicting outcome for stroke patients received thrombolysis treatment. **(C)** Relative level of NLR in good outcome group compared to poor outcome group for stroke patients not received thrombolysis treatment. ****P<0.0001, Mann-Whitney U test. **(D)** ROC of NLR in predicting outcome for stroke patients not received thrombolysis treatment.
